# Factors associated with instrumental support in transitional care among older people with chronic disease: a cross-sectional study

**DOI:** 10.1186/s12912-022-01014-w

**Published:** 2022-08-22

**Authors:** Min Cui, Jianing Hua, Xiaoliu Shi, Wenwen Yang, Zihan Geng, Xiangyun Qian, Guiling Geng

**Affiliations:** 1 School of Medical, Nantong University, No19, Qixiu Road, Chong Chuan District, Nantong, Jiangsu Province 226001 China; 2 Affiliated Hospital of Jiangnan University, 1800 Lihu Avenue, Wuxi, Jiangsu Province China; 3 Affiliated Hospital of Nantong University, 20 Xisi Road, Chongchuan District, Nantong, Jiangsu Province China; 4 Affiliated Nantong Hospital 3 of Nantong University, No. 60 Qingnian Zhong road, Chongchuan District, Nantong, 226001 Jiangsu China

**Keywords:** Instrumental support, Older adults, Transitional care

## Abstract

**Background:**

Instrumental support, which is defined as practical, tangible, and informational assistance extended to patients, is crucial for older people in transition. However, little is known about instrumental support in transitional care. Thus, the aim of this study was to evaluate the instrumental support of older people in transitional care.

**Methods:**

This cross-sectional study was conducted using the Questionnaire of Instrumental Support in Transitional Care (QISCT) to collect data from 747 older people in China from September to November 2020. Survey items consisted of a sociodemographic characteristics questionnaire and the QISCT. Multiple regression analyses were conducted to examine the association between independent variables and the QISCT scores.

**Results:**

The total score of the QISCT was 39.43 (± 9.11), and there was a significant gap between the anticipated support and received support. The satisfaction of instrumental support was low. Multiple regression analyses showed that educational level, the number of intimate relationships, monthly family income, monthly costs of transitional care, diabetes, and chronic obstructive pulmonary disease were associated with instrumental support in transitional care.

**Conclusions:**

To cope with the burden caused by chronic disease, the government and transitional care teams should establish a demand-oriented transitional care service model and pay more attention to helping older people obtain adequate and satisfactory instrumental support.

## Background

China is the country with the largest elderly population, and it is a place where the ageing of the population is taking place most rapidly [1]. The number of people aged 80 years or older is on track to reach 115 million by 2050 [2]. Ageing is the primary driver of many chronic diseases, such as heart disease, stroke, diabetes, and hypertension [3–5]. It was reported that up to 75.8% of adults aged 60 years or older had at least one chronic disease [6]. Moreover, more than 40% of them had multiple diseases [7]. These long-term diseases have become the leading causes of death and disability worldwide [8]. The life pattern of older patients is characterized by high rates of hospital readmission, frequent transitions between different locations, and the long-term involvement of patients, families, and health care providers [9]. Readmissions pose burdens to health care systems and patients, not only in regard to subjective well-being but also to the economic impact of costly hospitalizations [10]. To reduce these burdens, transitional care has been adopted in numerous countries.

Defined by the American Geriatrics Society, transitional care refers to a set of actions designed to ensure the coordination and continuity of health care as patients transfer between different locations or different levels of care within the same location [11]. The transitional care model (TCM), which is a multicomponent, nurse-led intervention, features a hospital-to-community dwelling intervention with nine components [12]. Transitional care teams composed of multidisciplinary members play an important role in the TCM. Meanwhile, transitional care units (TCUs) have been widely used to provide psychosocial support and meaningful modality education for patients in many countries [13]. This approach is a cost-effective quality improvement intervention because it can make full use of human and material resources [14]. Several studies have demonstrated the effectiveness of the TCM and TCUs in reducing all‐cause mortality, rates and length of readmission, and hospitalization costs [14–16].

In China, the primary health-care system has many shortfalls, including insufficient training for practitioners, insufficient continuity of care, and a shortage of integration between public health services and clinical nursing [17]. Therefore, transitional care has been mainly provided by clinical nurses rather than community nurses through follow-up phone calls and home visits [18]. For both patients and providers, effective transitional care programs are expensive in terms of time, cost, and personnel [19]. Additionally, different from developed countries, the cost of transitional care is not a part of the basic medical insurance system in China, which leads to a greater financial burden being placed on older people. With limited funds and human resources, the obstacle to improving the quality of transitional care is the lack of financial assistance, staff support, and services [20, 21].

The role of social support in the psychosocial transition of patients with chronic disease has been a hotspot in health-care research since the 1990s. Social support is a complex construct [22] and can be categorized into different types (e.g., instrumental, appraisal, emotional support) and forms (e.g., behaviours, perceptions). Dividing social support into emotional support and instrumental support is the most common classification. Emotional support includes the provision of care, love, and concern, thereby reflecting the activities that are regarded as basic elements of noninstructional counselling [23]. Instrumental support typically includes tangible or practical forms of support, such as task assistance, funds, and direct interventions, which can be used to directly alleviate strains [24]. In this study, instrumental support was defined as the practical, tangible, and informational assistance extended to patients.

Considering the lack of staff support and financial burden in transitional care, we decided to investigate the role of instrumental support in transitional care and to assess the older adult's perception of the amount of support they received. However, most of the previous studies that measured instrumental support used the social support scale or a part of it [25], and some of these studies lack reliability and validity [26]. Meanwhile, few scales have been specifically developed for instrumental support in transitional care. Therefore, we constructed a questionnaire of instrumental support in transitional care and used it to conduct a survey. Knowledge gained by use of this questionnaire with a targeted population may include opportunities to (1). Analyze the gaps between anticipated support and received support in regard to instrumental support; (2). Explore the factors influencing instrumental support; (3). Provide advice and guidance for the government and transitional care teams to promote the development of transitional care.

## Methods

### Study design

The cross-sectional survey used a convenience sample of older people from two communities and three public metropolitan hospitals in Jiangsu, China, from September to November 2020.

### Participants

The survey population included older people who were readmitted to the hospital and those transitioning from hospital to home. The criteria for selecting participants were (1) being aged 60 or older; (2) having a self-reported diagnosis of chronic disease; (3) receiving transitional care within three months; (4) being able to read, write or communicate; and (5) volunteering to participate in the research. We excluded those with severe cognitive impairment (MMSE ≤ 9) and those in intensive care.

To make the linear regression model stable, the minimum sample size should be 10–20 times the number of variables [27]. Considering the 14 variables in this study, the sample size was calculated as a minimum of 280. A 20% noncompletion rate increased the sample size to 336.

### Measures

#### Sociodemographic characteristics questionnaire

The sociodemographic characteristics of the participants included age, gender, marital status, education level, occupation, primary caregivers, the number of intimate relationships (who provides care or support at least once a week), monthly family income, medical insurance, monthly costs of transitional care, chronic disease (e.g., heart disease, hypertension, stroke, diabetes, chronic obstructive pulmonary disease), and risk factors (i.e., alcohol, smoking, and exercise).

#### The questionnaire of instrumental support in transitional care

The Questionnaire of Instrumental Support in Transitional Care (QISCT), which is based on social support theory and expectations and practical support theory, was constructed by our research group. We initially constructed the questionnaire through qualitative interviews held with older people and expert consultations. To further revise and improve the questionnaire, 180 older people were invited to participate in a preliminary survey. Through exploratory factor analysis, three factors were extracted (e.g., anticipated support, received support, and support satisfaction), and the cumulative contribution rate was as high as 69.128%. The Cronbach’s alpha of the QISCT was 0.827, and the test–retest reliability was 0.818, which indicated that the QISCT was reliable [28].

The QISCT has twelve items and three dimensions, including received support, anticipated support, and support satisfaction. Each dimension includes four aspects: service support, ways to receive support, staff support, and financial support. We were able to assess whether the four aspects of support were anticipated by, received by, or met the satisfaction of older people. By comparing what they anticipated with what they received, instances of unmet instrumental support can be found. The satisfaction of support was also measured by the questionnaire.

Scoring of the instrument: Items from 1 to 8 adopt the cumulative integral method (i.e., each item had the related options, one option counted for one point); the other items use a 5-point Likert scale ranging from 1 (strongly disagree) to 5 (strongly agree). The total score ranges from 8 to 120. A higher score indicates a higher level of instrumental support.

### Data collection

From September to November 2020, participants were recruited from two community health centers and three public metropolitan hospitals in Jiangsu through the referrals of nurses and word of mouth. Research assistants explained the aims and procedures of the study to eligible participants and asked them to sign informed consent forms. Then, participants were asked to complete the questionnaires. For those who had difficulty completing the questionnaire independently, researcher assistants read all the items and completed the questionnaire based on the response of participants.

### Data analysis

To ensure accuracy, all data were double-entered and checked. The analyses were performed with SPSS 21.0. The participants’ sociodemographic characteristics were described in terms of number and percentage. The QISCT score was described by the mean and standard deviation. To compare QISCT scores between different groups, we used the independent-sample *t* test and ANOYA test (*p* < 0.05). Finally, the factors associated with instrumental support were assessed using multiple regression analysis. As the traditional truncation of *p* < 0.05 could fail to identify known clinically important variables [29], we propose *p* < 0.3 as an appropriate threshold for selecting variables. Additionally, we used the chi-square test to compare the differences between anticipated support and received support (*p* < 0.05).

## Results

A total of 760 older people were recruited for the research through convenience sampling; however, nine participants did not complete the survey because of scheduling conflicts with other events. Four invalid questionnaires with misfiled questions were excluded. Finally, 747 valid questionnaires remained. The effective recovery rate was 98.2%.

### Participant characteristics

Of all the subjects, 339 were female (45.4%), 160 were over the age of 80 (21.4%), and 609 had a spouse (81.5%). Only 89 (11.9%) had more than five intimate relationships. The most common monthly income range was 2000–4000 yuan, and the monthly costs of transitional care for 153 people exceeded 1000 yuan. Table 1 describes the characteristics in detail.

**Table 1:** Socio-demographic characteristics of older people with chronic disease (*N* = 747)

**Variables**		**N (%)**	**QISCT** **(M ± SD)**	***t/F***	***p***
Age	60–69	291 (39.0)	38.91 ± 8.99	2.81	0.061
	70–79	296 (39.6)	40.39 ± 9.52		
	≥ 80	160 (21.4)	38.59 ± 8.43		
Gender	male	408 (54.6)	39.72 ± 9.06	0.957	0.339
	female	339 (45.4)	39.08 ± 9.18		
Marital status	have a spouse	609 (81.5)	39.57 ± 9.26	-0.862	0.389
	no spouse	138 (18.5)	38.83 ± 8.45		
Educational level	primary school or below	380 (50.8)	39.50 ± 9.44	3.792	0.010
	junior high school	196 (26.2)	38.10 ± 9.75		
	high school	120 (16.1)	39.98 ± 7.85		
	junior college or above	51 (6.8)	42.71 ± 5.28		
Occupation	unemployed	108 (14.5)	39.03 ± 9.04	1.077	0.367
	farmer	222 (29.7)	39.96 ± 9.62		
	worker	213 (28.5)	38.59 ± 9.63		
	company worker	113 (15.1)	40.64 ± 8.06		
	government worker	91 (12.2)	39.75 ± 7.78		
The number of intimate relationships	0	25 (3.3)	27.10 ± 6.50	13.70	< 0.001
	1–2	328 (43.9)	38.50 ± 9.33		
	3–5	305 (40.8)	39.60 ± 8.83		
	> 5	89 (11.9)	40.85 ± 9.20		
Primary caregivers	none	65 (8.7)	41.23 ± 8.89	1.176	0.318
	spouse	305 (40.8)	38.92 ± 8.85		
	child	308 (41.2)	39.55 ± 9.55		
	others	69 (9.2)	39.45 ± 8.42		
Monthly family income	< 2000	152 (20.3)	38.30 ± 9.01	1.504	0.186
	2000–4000	174 (23.3)	40.80 ± 9.58		
	4001–6000	125 (16.7)	38.94 ± 8.07		
	6001–8000	105 (14.1)	39.53 ± 8.94		
	8001–10000	70 (9.4)	40.14 ± 10.06		
	> 10000	121 (16.2)	38.88 ± 9.04		
Monthly expenses on transitional care	< 500	398(53.3)	38.40 ± 9.01	9.773	< 0.001
	500–1000	196(26.2)	38.31 ± 9.27		
	> 1000	153(20.5)	41.60 ± 9.53		
Medical insurance	Basic medical insurance for urban residents	332 (44.4)	38.19 ± 8.38	4.178	0.006
	Basic medical insurance for urban workers	147 (19.7)	41.10 ± 9.20		
	New type of rural cooperative medical care	233 (31.2)	40.06 ± 9.78		
	others	35 (4.7)	40.03 ± 9.62		
Diagnosis of chronic diseases	hypertension	435 (58.2)	39.33 ± 9.19	0.366	0.715
	diabetes	274 (36.7)	40.27 ± 9.26	-1.930	0.054
	stroke	99 (13.3)	39.91 ± 7.84	-0.562	0.575
	heart disease	81 (10.8)	40.28 ± 9.08	-0.893	0.372
	chronic obstructive pulmonary disease	67 (9.0)	40.78 ± 8.78	-1.268	0.205

### Factors associated with instrumental support in transitional care

Based on univariate analysis, many factors (*p* < 0.3) served as dependent variables in the multiple linear regression model. The results of multiple regression showed that educational level, the number of intimate relationships, monthly family income, monthly costs of transitional care, diabetes, and chronic obstructive pulmonary disease all had a significant relationship with instrumental support in transitional care, which explained 22.5% of the variance. Compared with those with primary school or lower qualifications, the level of instrumental support for older people with junior college or higher qualifications was higher. Older people with a monthly income of 2000–4000 yuan had higher QISCT scores than those with a monthly income of less than 2000 yuan, and the participants who spent more than 1000 yuan a month on transitional care had higher QISCT scores than those who spent less than 500 yuan a month. The results also showed that the QISCT scores of older people without intimate relationships were significantly lower than those with intimate relationships. In addition, older people with diabetes or COPD had higher QISCT scores than those of their counterparts. Table 2 shows the results in detail.

**Table 2:** Factors associated with instrumental support in transitional care (*N* = 747)

**Variables**		**B**	**SE**	**β**	***t***	***p***
Constant		24.779	1.700		14.580	< 0.001
Educational level	primary school or below (reference)					
	junior high school	2.088	0.743	0.225	2.810	0.005
	high school	0.799	0.901	0.086	0.886	0.376
	junior college or above	5.811	1.302	0.627	4.462	< 0.001
The number of intimate relationships	0 (reference)					
	1–2	6.215	1.763	0.671	3.525	< 0.001
	3–5	7.035	1.784	0.759	3.943	< 0.001
	> 5	9.612	1.946	0.937	4.939	< 0.001
Monthly income of the family	< 2000 (reference)					
	2000–4000	3.819	0.958	0.412	3.988	< 0.001
	4001–6000	1.725	1.048	0.186	1.647	0.100
	6001–8000	1.228	1.145	0.132	1.072	0.284
	8001–10,000	0.820	1.272	0.088	0.645	0.519
	> 10,000	0.280	1.081	0.030	0.258	0.796
Monthly expenses on transitional care	< 500 (reference)					
	500–1000	0.020	0.732	0.002	0.027	0.979
	> 1000	2.817	0.813	0.304	3.464	< 0.001
Diabetes	No (reference)					
	Yes	4.979	0.639	0.537	7.796	< 0.001
chronic obstructive pulmonary disease	No (reference)					
	Yes	3.173	1.076	0.342	2.950	0.003
*F(p)*	10.700	(< 0.001)				
Adjusted *R*^2^	0.225					

### Degree of instrumental support in transitional care

The mean total score of the QISCT was 39.43 (± 9.11), with a range of 20–79. The mean score for anticipated support was 12.67 (± 5.90), for received support was 10.29 (± 4.20), and support satisfactory score was 16.47 (± 2.98).

#### Dimensions of received support and anticipated support

The top 5 received support and anticipated support were nursing support, doctor support, disease guidance, rehabilitation guidance, and exercise guidance. There were significant differences between received support and anticipated support in many aspects, except for disease guidance, nursing support, and financial support from friends. Table 3 shows the gaps in detail.

**Table 3:** Gaps between received support and anticipated support

Items		Received support (%)	Anticipated support (%)	*χ*-value^2^	*p*-value
Service support	Disease guidance	67.5	68.4	0.226	0.634
	Rehabilitation guidance	30.3	42.8	43.2	< 0.001
	Exercise guidance	28.6	41	29.8	< 0.001
	Living guidance	16.2	29.7	46.1	< 0.001
	Safety instructions	10	19.9	36.5	< 0.001
	Psychological guidance	7.1	17.1	37.8	< 0.001
	Guidance on prevention of complications	7	23.8	85.9	< 0.001
	Change dressing for wound ostomy	1.5	2.8	4.5	0.034
	Replace and flush lines	0.9	2.9	8.52	0.004
Ways to get support	Paper-based materials	22.9	29.5	10.4	0.001
	Television	13.7	35.9	123	< 0.001
	Health lecture	11.0	25.7	78.2	< 0.001
	Home visits nursing	8.8	34.4	156	< 0.001
	WeChat, QQ	7.8	19.0	50.7	< 0.001
	Administration home patient	1.6	6.6	28.2	< 0.001
Staff support	Doctor	66.0	74.6	18.4	< 0.001
	Nurse	77.4	79.8	1.46	0.227
	Community worker	7.9	12.0	8.26	0.004
	Caregiver	5.6	12.7	30.4	< 0.001
	Volunteer	4.7	13.9	54.4	< 0.001
	Physical therapist	3.7	23.6	132	< 0.001
	Nutritionist	2.7	22.4	128	< 0.001
	Pharmacist	1.3	16.6	101	< 0.001
	Counselor	0.3	8.7	61.0	< 0.001
Financial support	commercial health insurance	6.6	11.9	26.2	< 0.001
	Long-term Care Insurance	3.2	20.9	113	< 0.001
	Impoverished rescue	4.0	6.2	4.89	0.027
	Help from friends	3.9	5.1	1.31	0.253
	Help from religious and social groups	0.5	6.4	38.5	< 0.001
	Subsidies for hypertension and diabetes	1.9	4.7	8.51	0.004

#### The satisfaction of instrumental support

**Fig. 1:**
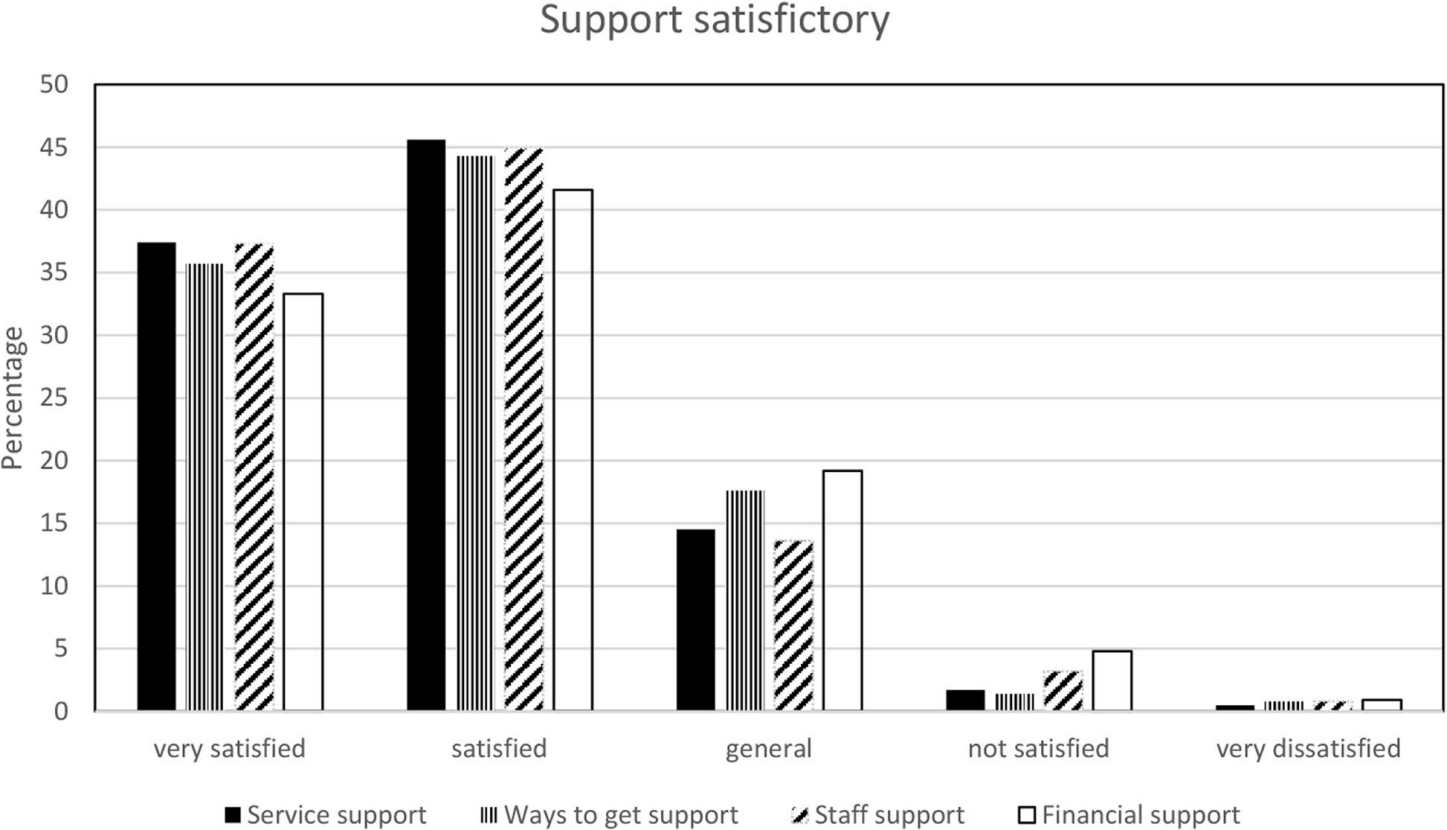
The satisfaction of instrumental support in transitional care

Most of the participants were satisfied with the level of instrumental support in transitional care. Compared with service support and staff support, the satisfaction level with financial support and the ways to get support were lower. Figure 1 shows the satisfaction of instrumental support.

## Discussion

This study explored the instrumental support situation for older people with chronic disease in transitional care by utilizing the QISCT. The results illustrated that the level of instrumental support in transitional care was low.

### Gaps between received support and anticipated support

Our results showed that nearly half of the older people did not receive guidance on safety, living, psychology or the prevention of complications they anticipated, and many older people’s needs for therapists, nutritionists, volunteers, pharmacists, and psychological counsellors were unmet. They were fully provided with the support of disease guidance and nursing support. Transitional care teams are dominated by doctors and nurses who focus on health education and management of disease [12] and lack multidisciplinary talent, such as that possessed by nutritionists, volunteers, pharmacists, and psychological counsellors [30, 31]. Hence, the government should expand transitional care teams and establish a demand-oriented transitional care service model to meet the diversified needs of older people.

Regarding the ways in which to obtain instrumental support, 34.4% of the participants anticipated home visits, while only 8.84% received home visits. Transitional care teams have many difficulties in addressing the need for home visits, including the lack of a supervision system and well-qualified caregivers [32]. Coordinating various departments to improve personnel training, supervision and charging systems is an effective way to promote the long-term development of home visits [33]. The needs for health lectures and online platforms were also unmet because of the existence of false information and digital gaps [34, 35]. The accuracy of information was emphasized as a key to ensuring transitional care [36]. Therefore, it is urgent for transitional care teams to establish a reliable network platform and carry out credible TV education and health lectures.

Regarding the aspect of financial support, the widest gap was seen in long-term care insurance. Long-term care insurance can provide care security and financial compensation for chronically incapacitated people, which has been shown to reduce inpatient expenditure and the length of stay in hospital. However, many participants were not satisfied with the performance and outcomes of their long-term care policy [37]. There were also significant gaps between received support and anticipated support in commercial medical insurance, subsidies for hypertension and diabetes, and nonofficial subsidies. Researchers have called for introducing a reimbursement mechanism or medical insurance related to transitional care to promote older people receiving more financial subsidies.

Although there were many significant gaps between received support and anticipated support, the majority of subjects were satisfied with instrumental support. For older adults, instrumental support was mostly provided by doctors or nurses who can provide professional services and advice, so they were satisfied with the support they received. Also, influenced by traditional culture, Chinese elderly were more tolerant [38], so they were likely to accept their situation and rarely expressed their dissatisfaction to others.

### The total score of instrumental support influenced by many factors

Our results illustrated that instrumental support was significantly associated with educational level, the number of intimate relationships, monthly family income, monthly costs of transitional care, diabetes, and chronic obstructive pulmonary disease.

Multiple linear regression results showed that educational level and the number of intimate relationships had positive effects on instrumental support in transitional care. Education was a protective factor for healthy behaviour, which was associated with older people's health literacy and self-management [39]. In addition, people with a higher level of education may have a comprehensive perception of things, which means that they can assess physical risk factors more effectively [40] and solve health problems in multiple ways. The number of intimate relationships was a factor associated with instrumental support. Many studies have emphasized the critical role of social networks in health-related behaviours, mental health, and social support [41]. Stoller reported that spouses and children are the primary providers of instrumental support for older people. In contrast, the level of instrumental support is higher among people whose social network centre is comprised of friends [42]. The diversity of one’s social network is significant to the quality of one’s instrumental support; thus, transitional care teams should pay more attention to socially isolated people.

The economy was found to have a significant impact on instrumental support in transitional care. Chronic disease causes an economic burden on older people. In a recent report, researchers estimated that the cost of informal caregiving for people with cardiovascular disease would double from 2015 to 2035, which would represent 11% of medical and production costs. Consequently, older people with higher incomes are more likely to receive diverse support from informal caregivers, and those who spend much more money on transitional care can receive adequate support. Therefore, in further studies, we should focus on the financial burden of older people and those who spend less money on transitional care to determine whether they receive enough support.

Among older people with chronic diseases, those with diabetes and chronic obstructive pulmonary disease had higher levels of instrumental support. Transitional care modes have been widely used in diabetes and chronic obstructive pulmonary disease [43, 44], and the American Diabetes Association (ADA) has presented guidelines for the care of patients during the discharge process. Therefore, with a decline in physical function, the anticipated and received levels of support for older people increase. In addition, a survey reported that during the postdiagnosis programming period, friends and neighbours provide plenty of instrumental support to people with diabetes [45]. However, the instrumental support provided for the older adults with other chronic diseases is not enough, which should be the concern of the transitional care teams.

## Limitations

This study has several limitations. First, this is a cross-sectional study, which precludes us from assigning directional causality relationships among variables. Second, to ensure the validity of the data collected, we excluded patients with severe cognitive impairment. However, the needs of these excluded patients are often unmet. We did not make a distinction between people coming from the community and hospitals. Third, respondents tended to give favourable rather than unfavourable evaluations; thus, it was hard for us to know their honest opinions. In future studies, we will continue to investigate why patients are satisfied with instrumental support versus dissatisfied through in-depth interviews.

## Conclusions

Currently, the level of instrumental support for older people with chronic disease in transitional care is generally low. Older adults with diabetes and chronic obstructive pulmonary disease have a higher level of instrumental support in transitional care. Our results suggest that educational level, the number of intimate relationships, monthly family income, and monthly expenses for transitional care are associated with instrumental support in transitional care. Prospective long-term follow-up studies are necessary to understand these changes in instrumental support. Our results indicate that older people have low access to financial support and many instrumental support requirements are unmet, so the government should improve fiscal support policies and establish a demand-oriented transitional care service model to meet the diversified needs of older people. In addition, the way for older adults to obtain instrumental support is relatively single, transitional care teams should establish a reliable network platform and carry out credible TV education to provide adequate instrumental support for older people.

## Data Availability

The datasets used and analyzed during the current study available from the corresponding author on reasonable request.

## Abbreviations

QISCT: Questionnaire of Instrumental Support in Transitional Care
TCM: Transitional Care Model
TCU: Transitional Care Unit
ADA: American Diabetes Association
